# Contrast-enhanced ultrasonography findings of LAMNs with peritoneal and splenic metastases: a case report and literature review

**DOI:** 10.3389/fonc.2023.1238042

**Published:** 2023-09-25

**Authors:** Mei Chen, Shengmin Zhang, Youfeng Xu, Xiupeng Jia, Yijiu Shi

**Affiliations:** ^1^Department of Ultrasonography, Ningbo First Hospital, Ningbo University, Ningbo, China; ^2^Department of Histology, Ningbo First Hospital, Ningbo University, Ningbo, China; ^3^Department of Surgery, Ningbo First Hospital, Ningbo University, Ningbo, China

**Keywords:** mucinous neoplasms, appendix, ovarian neoplasms, spleen, ultrasonography

## Abstract

Low-grade appendiceal mucinous neoplasms (LAMNs) are rare appendiceal tumors that are primarily diagnosed using computed tomography(CT) enhancement and magnetic resonance imaging (MRI). Herein, we report the sonographic features, especially for contrast-enhanced ultrasound (CEUS), of a 70-year-old female with an unusual LAMN metastasizing to the peritoneum and spleen. The patient had a right pelvic mass 2 days prior to presentation. Two-dimensional (2D) ultrasound revealed a mixed cystic-solid mass in the right lower abdomen and spleen parenchyma; CEUS showed heterogeneous enhancement in both areas, suspected to be a mucinous mass. CT enhancement and MRI findings revealed concurrent findings. Histopathologically, LAMN lesions were confirmed in the appendix, spleen, and peritoneum of the specimens obtained during exploratory laparoscopy. No recurrences were reported at three years postoperatively. LAMN lesions may metastasize to abdominal organs, and imaging examinations are essential for diagnosis. This study presents major ultrasonography and CEUS findings for the diagnosis of LAMNs.

## 1 Introduction

Low-grade appendiceal mucinous neoplasms (LAMNs) are rare, comprising only 0.2%–0.3% of appendiceal neoplasms (1). Histopathologically, there are three types of myxoid tumors of the appendix: myxadenoma, LAMN, and adenocarcinoma (2). Although a benign adenoma has low malignancy rates, a LAMN may demonstrate specific pathological features similar to mucinous tumors that involve organs outside the appendix with uncertain malignancy potential (3). Pathologically, LAMN is confined to the appendiceal mucosa and does not invade the muscularis mucosae (4). LAMNs are high-risk tumors with low recurrence rates (4) and are known to invade adjacent organs in the right lower abdomen. However, they rarely metastasize to the spleen and peritoneum (1).

The clinical features of LAMNs were not pathognomonic. It usually presents with symptoms of acute-onset appendicitis caused by appendicular luminal obstruction by a neoplastic mass. Some patients may present with lower abdominal pain, while others may be asymptomatic; this discovery may be an incidental finding (5). Due to the inconsistency in the clinical manifestations of LAMNs, imaging examinations are extremely important to avoid misdiagnosis. Computed tomography (CT) and magnetic resonance imaging (MRI) are highly sensitive modalities, but the examination is expensive, and the equipment is not widely available in smaller hospitals. Ultrasonography is a simple and economical imaging technique that can be performed easily. Furthermore, contrast-enhanced ultrasound (CEUS) can reveal the microcirculatory blood flow in different tissues to improve diagnostic accuracy (6).

Herein, we present a rare case of LAMN with peritoneal and splenic metastases diagnosed pathologically after surgery in an elderly female. Two-dimensional (2D) and CEUS findings of the appendix and spleen have been summarized, which may be used for diagnosing LAMNs.

## 2 Case presentation

**Figure 1:**
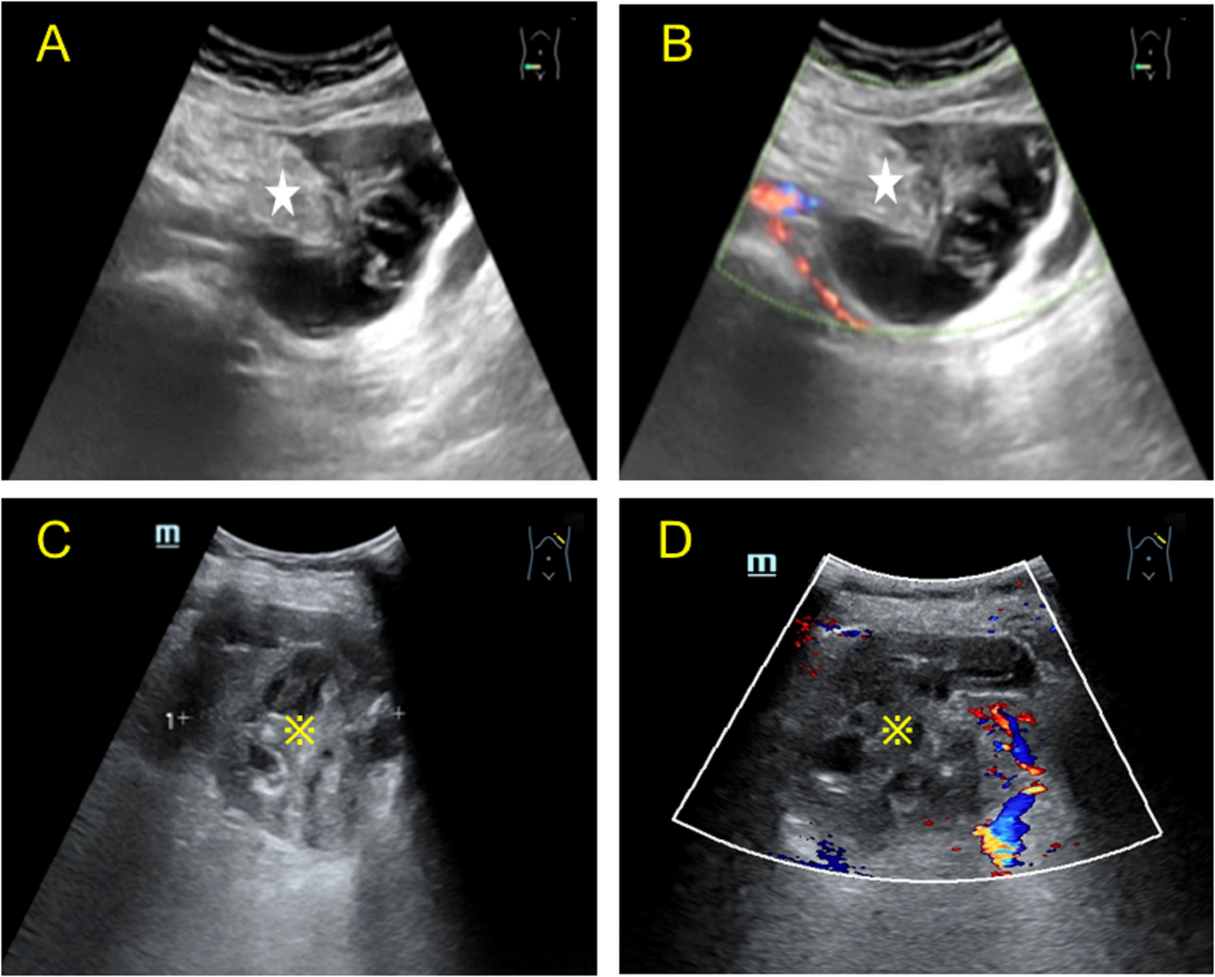
Two-dimensional Color Doppler images of the lesion in the right lower abdomen and spleen obtained by transabdominal ultrasound. **(A)** Longitudinal view of the transabdominal ultrasound scan of the right lower abdomen, showing a mixed cystic and solid echo. The solid part (asterisk) was eccentric, most of the surrounding tissue was cystic, and a radial stripe light band was seen in the dark area connected to the solid part. **(B)** Color Doppler of the mass showed minimal linear blood flow in the solid part; heterogeneous echogenicity was observed in the right adnexa, mainly anechoic around some hyperechoic echogenicity in the center. **(C)** Transabdominal ultrasound of the spleen showing a mixed cystic and solid mass (※) in the left hypochondrium, with irregular dark liquefied areas septate (left-right diameter of the mass: 7.52 cm). **(D)** Color Doppler image showing sparse blood flow in the solid part and no significant blood flow distribution in the cystic part.

**Figure 2:**
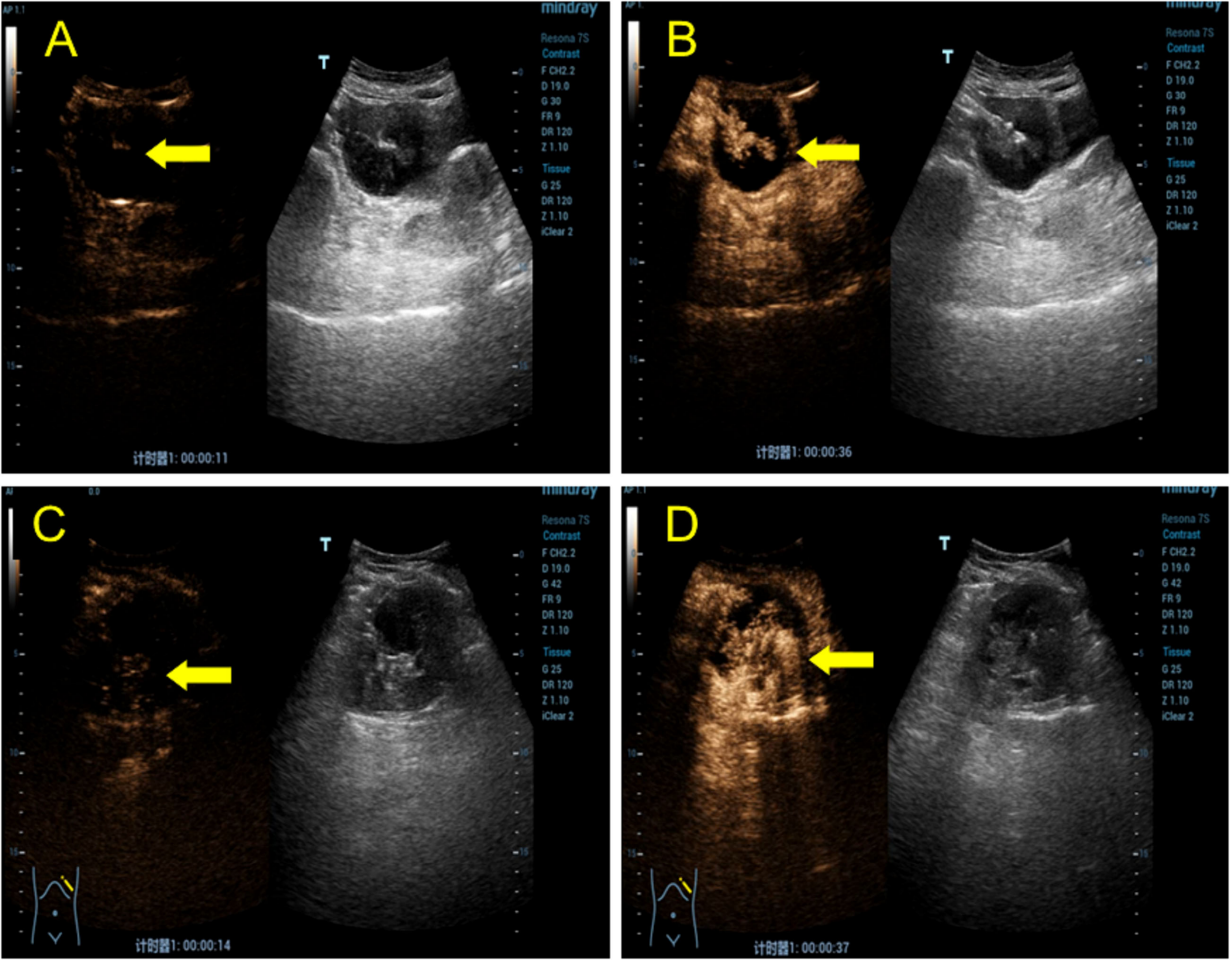
CEUS shows a lesion in the right lower abdomen and spleen. **(A)** In the transabdominal contrast-enhanced ultrasound examination, the solid capsular part of the mass in the right lower abdomen began to perfuse at 11 s (yellow arrow). **(B)** Isoenhancement is seen in the center (yellow arrow), and no contrast perfusion is observed in the cystic portion. **(C)** During the transabdominal contrast-enhanced ultrasound examination, the mixed mass of cystic and solid capsulated in the spleen began to perfuse at 14 s (yellow arrow). The contrast agent was unevenly distributed, with visible perfusion in the solid part but not in the cystic part. **(D)** The contrast agent entered the solid part (yellow arrow), which was isoenhanced, and the enhancement subsided at 37 s, which was earlier than that of the normal spleen tissue.

A post-menopausal (15 years ago) 70-year-old female was admitted to our hospital with a high tumor index and a pelvic mass detected two days ago. The patient had elevated CEA levels (15.3 ng/ml; reference range: 0 ng/ml–5 ng/ml). She had only one living child and had no relevant clinical complaints. Physical examination revealed painful swelling in the right abdomen, specifically in the right iliac fossa. There were no complaints or signs of fever, nausea, vomiting, constipation, melena, or hematochezia. Laboratory examination after admission showed the following findings: cancer antigen (CA)-125 (CA-125): 47.30 IU/ml (reference range: 0 IU/ml–35 IU/ml), cancer antigen CA-724: 15.5 IU/ml (reference range: 0–7 IU/ml), and Cancer antigen CA-199: 64.4 IU/ml (reference range: 0 IU/ml–39 IU/ml). No apparent abnormalities were observed in the routine blood and urine, hemagglutination, thyroid hormone, or sex hormone tests. Furthermore, gastroscopy with biopsy revealed chronic superficial gastritis with erosion (gastric antrum). Ultrasound of the lower abdomen revealed a mixed, and mainly cystic, mass with heterogeneous, measuring 6.5 cm × 4.2 cm at the right end and increased echogenicity in the center (4.5 cm × 2.1 cm) (**Figure 1A**). Color Doppler flow imaging (CDFI) revealed minimal flow in the center with a resistance index of 0.56 (**Figure 1B**). There was an incomplete splenic capsule with a mixed echo (size: 7.0 cm × 5.7 cm × 7.5 cm), with irregular morphology and an ill-defined border, obscuring differentiation from the normal splenic tissue (**Figure 1C**). However, CDFI showed little flow in the mixed echo area, except in the anechoic part (**Figure 1D**). To define the microvasculature of the mass, CEUS was performed, in which the agent was perfused within 11 s in the solid part of the mass in the right lower abdominal region (**Figure 2A**). The signal was equal to that of the intraperitoneal intestinal tract and regressed simultaneously but without any contrast agent perfusion in the anechoic part (**Figure 2B**). In the CEUS of the spleen, the solid part began to enhance at 14 s, and the contrast agent perfused with equal enhancement; the regression began at 37 s, which was earlier than that for the normal splenic tissue (**Figures 2C, D**). The CEUS images of the spleen and appendix had a common feature of coronal enhancement with central perfusion of the contrast agent, while the contrast agent did not perfuse in the non-echo part. Accordingly, we speculated that a mucinous lesion was present in the appendix with splenic metastases.

**Figure 3:**
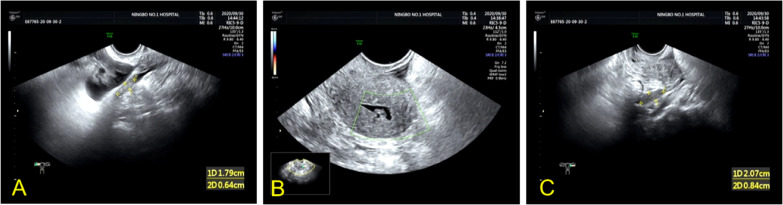
Transvaginal ultrasound images of the uterus and bilateral adnexa of the patient. **(A, C)** Atrophic right and left ovaries can be visualized. **(B)** TVU image revealing an atrophic uterus without intimal thickening.

**Figure 4:**
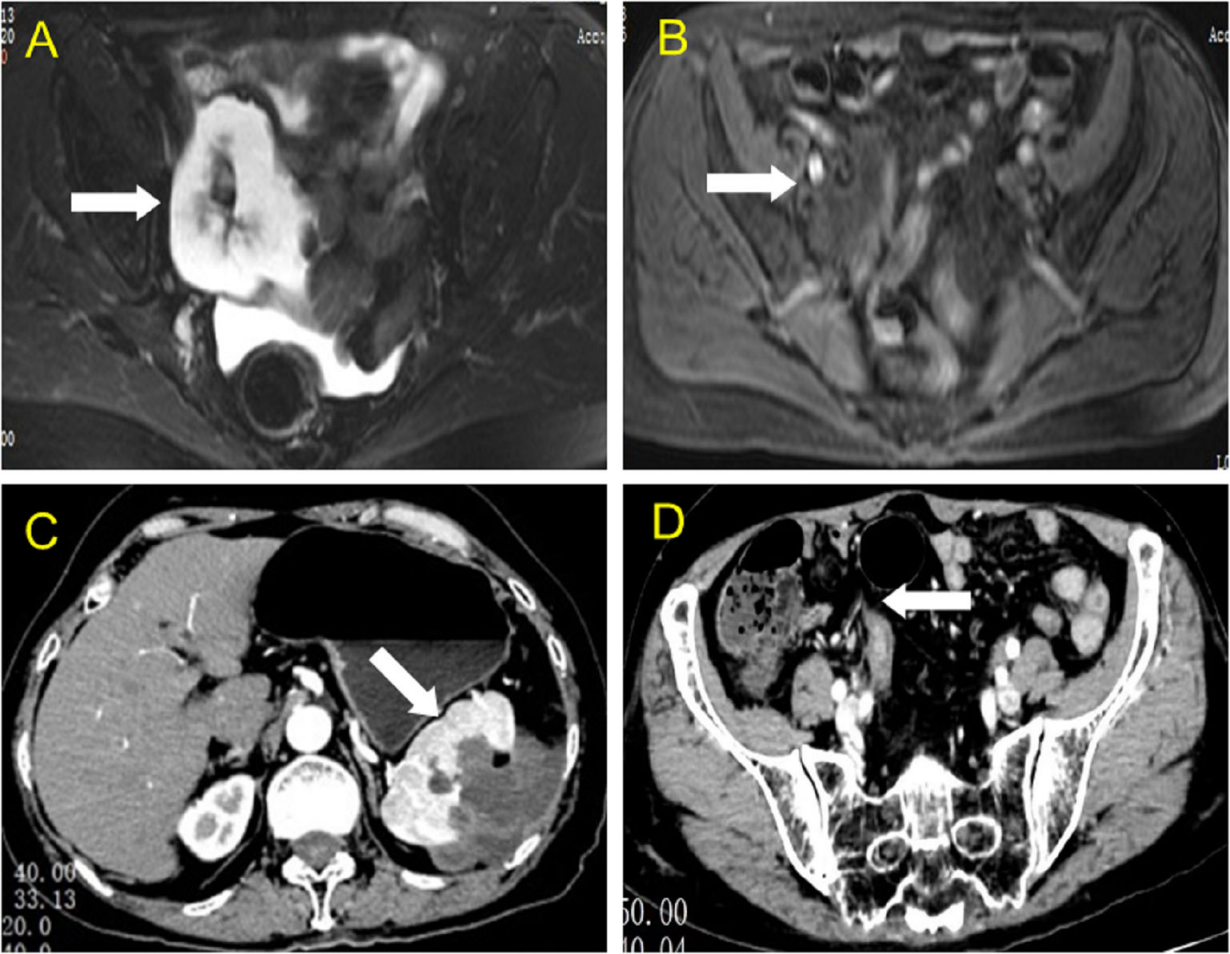
MRI and CT findings of the lesion in the right lower abdomen and spleen. **(A, B)** Axial view of the abdominal magnetic resonance scan revealed a slightly short T2 signal, a heterogeneous high signal in the diffusion-weighted image, and high signal intensity in the solid part (white arrow). **(C)** Axial view of contrast-enhanced computed tomography showing a multilocular cystic focus at the lateral splenic border (white arrow), which showed no significant enhancement after intracystic contrast perfusion, and intensity enhancement of the cyst wall after contrast perfusion. **(D)** Axial view of contrast-enhanced computed tomography revealed a mixed mass in the right lower abdomen, with significant enhancement in the central solid part.

Based on the preliminary findings of a mass in the right lower abdomen of a female patient, a transvaginal ultrasound scan was performed to visualize the uterus and bilateral adnexa, which revealed an atrophied uterus without intimal thickening (**Figure 3B**), and both ovaries could be visualized (**Figures 3A, C**). Furthermore, MRI enhancement revealed a mixed cystic and solid shadow on the right side of the pelvis, with a short signal on T2WI and a heterogeneous high signal on diffusion-weighted imaging (**Figures 4A, B**). The contrast-enhanced CT scan revealed that the solid part of the mass was significantly enhanced, and the periphery was connected to the omentum (**Figure 4D**), suggestive of pelvic inclusion effusion or a teratoma. The contrast-enhanced CT scan demonstrated a multilocular cystic focus at the lateral splenic border, which showed no significant enhancement in the intracystic region or cystic wall after contrast addition (**Figure 4C**). These findings indicated a diagnosis of LAMN.

**Figure 5:**
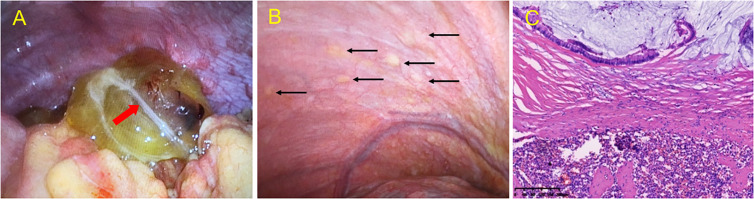
Intraoperative view during exploratory laparoscopy and microscopic histopathological examination of lesions. Intraoperative view during exploratory laparoscopy revealed a yellow cystic mass in **(A)** the appendix region, with a milky white flocculent light band (red arrow) in the cyst. **(B)** Intraoperative view during exploratory laparoscopy revealing multiple yellow vesicular nodules (black arrow) in the peritoneum. **(C)** Hematoxylin and eosin (HE) staining of a low-grade mucinous tumor of the spleen, involving both the parenchyma and exterior of the splenic capsule and low-grade mucinous epithelial cells in the mucus lake. Original magnification: ×10.

Eventually, the patient was prepared for laparoscopic exploratory surgery, which revealed a yellow-colored 7.0 cm cystic mass in the right lower abdomen (**Figure 5A**). The splenic tumor (measuring 5.0 cm × 4.0 cm) partially covered the surface, invading the splenic tissue. Multiple yellow vesicular nodules were found in the peritoneum (**Figure 5B**), and histopathological examination of the biopsy specimen revealed a low-grade mucinous tumor in the appendix, involving the entire appendix and surrounding fat tissue (**Figure 5C**), along with invasion of the spleen and peritoneum. Eleven perisplenic lymph nodes showed reactive hyperplasia, and the peritoneal nodules and small mesenteric nodes were also diagnosed as LAMN.

Since was many peritoneally disseminated lesions were observed during the operation, complete resection of all lesions was not possible. After discussion with the patient’s family, the patient was administered hyperthermic intraperitoneal chemotherapy (HIPEC) after resecting the appendiceal and splenic lesions. There were no instances of recurrence during the regular 6-month follow-up over three years postoperatively.

## 3 Discussion

The incidence of LAMN has been reported to double in males and females (7). Some patients may present with right lower abdominal pain, and they can be misdiagnosed as having an adnexal mass or acute appendicitis before the operation (8). Therefore, pelvic masses in females should be evaluated carefully to determine if they are of gynecological origin or a neoplasm. On physical examination, the mass may be palpable in some patients either when it is significantly large or when the patient has less abdominal fat. Imaging is required to confirm these findings, imaging examinations are required. Previous studies have summarized the characteristic findings of LAMN during different imaging examinations such as ultrasound, CT, and MRI (3, 6); however, the CEUS characteristics of LAMN have not been accounted for. While CT and MRI are considered more advantageous for the diagnosis of LAMN (9), the examination is expensive, and the equipment is not widely available in smaller hospitals.

Since splenic metastasis in LAMN is extremely rare, there is a paucity of relevant literature. Only eight cases originating from the appendix and one from the colon with splenic metastasis have been reported, all of which were diagnosed using CT (10). **Table 1** summarizes the clinical data and imaging findings of patients reported in the literature.

**Table 1:** Clinical features and imaging findings of patients with splenic metastases associated with mucinous appendiceal and colonic neoplasms.

Age (years)	Sex	Site of the primary tumor	Clinical features	Imaging findings	Pathology type
47	Male	Appendix	right inguinal hernia	CT: have extensive ascites and multiple spherical defects inside the splenic parenchyma	LAMN
38	Female	Appendix	abdominal pain	CT: a large intraparenchymal defect of the spleen	LAMN
38	Male	Appendix	acute appendicitis	CT: a defect in spleen	LAMN
29	Female	Appendix	Abnormal routine test	CT: an intraparenchymal splenic defect enlarging	LAMN
48	Male	Appendix	repairing of an umbilical hernia	CT: multiple peritoneal implants and a cystic an intraparenchymal mass in the spleen	LAMN
48	Male	Appendix	appendicitis	CT: an intraparenchymal splenic mass	LAMN
60	Male	Appendix	appendicitis	CT: a cystic intraparenchymal defect	MA
48	Female	Appendix	abdominal pain and uterine bleeding and large uterine fibroids and probable endometriosis	CT: an intraparenchymal splenic mass	LAMN
52	Female	Colon	bowel obstruction	CT: an intraparenchymal disease compatible with metastases	MA
45.3 (29–60)	5 Males 4 females	8 Appendix 1 Colon	3 appendicitis/2 hernia/2 abdominal pain/1 bowel obstruction/1 routine test	9 CT	7 LAMN/ 2 MA

CT, Computer Tomography; LAMN, Low-grade appendiceal mucinous neoplasm; MA, mucinous adenocarcinoma.

The patient’s age is at the time of initial diagnosis. (Derive from Cabanas J, et al. Tumori, 2006;92(2):104-12) (10).

Ultrasound is a non-invasive, repeatable, and accessible imaging method; hence, it is the preferred screening method for abdominal pain. In our study, ultrasound images were closely related to the pathology. Most patients have a mixed cystic and solid mass in the right lower abdomen on 2D ultrasound, with unclear flow on color Doppler. Some experts define mixed cystic and solid masses as onion changes on 2D ultrasound (10). These sonographic manifestations are due to edema and thickening of the appendiceal mucosa and the muscular and serosal layers (11); mucus accumulation in the appendiceal lumen is responsible for cystic expansion (11). This continuous mucus accumulation is visible as sonographic changes; the resultant increase in tension eventually leads to progressive rupture of the appendix (11). Determining the blood supply of tumors is essential to establish the structure of the mass; however, owing to the interference of breathing and depth limitations, it is difficult to truly reflect the blood supply of the mass using 2D ultrasound alone. Developments in contrast agents and ultrasound techniques have enabled the diagnosis of a deep abdominal mass with CEUS by visualizing the microvascular flow inside the mass. As clinical manifestations are related to pathology, patients may experience weight loss, appendicitis, nausea, and vomiting (12, 13). Furthermore, it is important to clarify whether a lower abdominal mass is of ovarian origin, and some authors have used a probe to palpate the mass and determine whether it is relatively movable over the ovary (14). If the mass moves in the opposite direction to the ovary, it is probably not of ovarian origin. In this patient, when we patched the mass with the probe, we could see relative motion with the right ovary. It was judged that the mass was not of ovarian origin but originated from other organs in the abdomen. Furthermore, a lesion in the right lower abdomen and inflammatory diseases such as a periappendiceal abscess should be ruled out. Owing to its convenience and economy, ultrasound is often recommended as the first imaging method for the diagnosis of appendiceal lesions and the preliminary judgment of its pathology. The ultrasonographic manifestations of appendiceal abscess are fluid collection (hypoechoic) in the appendiceal area, which could be a round mass with clear boundaries or irregular shapes with unclear boundaries. Ultrasonographic changes in the appendix tissue were observed in the mass, and interruption of the appendiceal wall was observed in some cases. On the other hand, the patient may have had abdominal pain, higher temperature, and abnormal blood test results.

The etiology and biological mechanism underlying LAMNs are unclear, and biological behavior does not necessarily correspond to the clinical course but depends on the clinical manifestations and disease severity (3). Intraoperative findings of LAMN are mostly confined to the abdominal organs, and extraperitoneal or lymph node metastases are rare. If the lesion is confined to the appendiceal wall, patients tend to have a good prognosis and low risk of recurrence. Interestingly, peritoneal dissemination of LAMN can lead to the formation of pseudomyxoma peritonei (PMP), which is the accumulation of mucinous ascites in the peritoneum or abdominal cavity (10). Metastasis of LAMN can involve other abdominal contents, especially the omentum, the right diaphragm, the right retrohepatic space, the Treitz ligament, and the left abdominal and pelvic cavity; distant metastasis is also possible. Association of LAMN with other intestinal tumors have also been reported, such as the long-term development of ulcerative colitis (15, 16). Low-grade appendiceal mucinous epithelial cells have low-grade atypia and are less invasive; hence, their prognosis is significantly different from that of high-grade PMP. Generally, low-grade PMP correrelates with low-grade myxoma, and high-grade PMP corresponds to mucinous adenocarcinoma; however, unanticipated occurrences are possible (17, 18). In our patient, low-grade mucinous tumor cells were found in the pathological sections of the appendix, peritoneum, and spleen obtained from the intraoperative biopsy. Presumably, the low-grade mucinous tumor cells broke through the spleen capsule and were transplanted from the appendix or peritoneum into the spleen. Some experts have labeled this as splenic pseudometastasis (10).

In our patient, the tumor marker CA-125 level was slightly higher than the normal range at the time of admission. However, it is unclear whether elevated CA-125 levels are specific to LAMN. It has been reported that in most cases this marker is elevated before surgery, which may be related to peritoneal dissemination (9). In addition, most studies consider CA-199 and CA-125 as good predictors of recurrence rather than diagnostic biomarkers (19). It is rare for LAMN to involve both the peritoneum and spleen (20); for metastasis to the spleen, mucin-producing epithelial cells may be trapped within the trabeculae of the splenic capsule, in congenital splenic clefts, or even in splenic microclefts caused by trauma during previous surgery (10). The mucus produced by these cells blocks their narrow entry point to destroy the tissues adjacent to the spleen, expanding into the splenic parenchyma and forming metastasis of the spleen, which is a possible mechanism of splenic lesions in our patient (10). Imaging findings of splenic metastasis in our patient became more evident over time. Considering the primary site of the PMP in the appendix, LAMN may metastasize to the left subphrenic space through the peritoneum, as the spleen is in the left upper abdomen, below the diaphragm, and covered by the upper peritoneum, resulting in continuous erosion of the spleen. However, hematogenous metastasis cannot be completely ruled out (10).

At present, there is no consensus on the surgical methods and modalities for the treatment of appendiceal mucoceles. For unruptured appendices, appendectomy and right hemicolectomy are recommended. However, cytoreductive surgery and HIPEC are recommended for patients with PMP rupture (21). Laparoscopic surgery is presumed to increase the risk of intraoperative rupture and mucus spread (10, 21); therefore, a definitive preoperative diagnosis of LAMN is crucial when choosing therapeutic methods.

In a previous study, LAMN of the appendix was diagnosed using 2D ultrasound which revealed cystic long tubular or ovoid lesions in the appendiceal region connected to the cecum (3). The inside of the lesion has an onion-peel appearance (representing lamellar mucin), a characteristic ultrasound finding of appendiceal mucinous neoplasms (10). The appendix wall can also be locally calcified to form a porcelain appendix, and calcification near the wall has a strong echo (22). This is the first case of LAMN with splenic metastasis diagnosed using contrast-enhanced ultrasonography (CEUS)—contrast filling in the central hyperechoic area (isoenhancement) and no contrast filling (non-enhancement)in the peripheral anechoic area of the right lower abdominal mass. The degree of enhancement is relative to that of the surrounding bowel. The isoenhanced part was the echo of the appendiceal tissue invaded by the LAMN and contained microvessels, and the contrast agent was reflected by microbubbles (6).The surrounding non-enhancing part was composed of mucin secreted by the tumor, suggestive of the characteristic “corona-like” appearance of LAMN on CEUS. CEUS of the spleen showed an anechoic area with a scallop-like outline and a grid shape, which may be related to LAMN metastasis in the spleen (23). The cystic wall has a microvascular component that can secrete cyst fluid (a mass accumulating mucus). CEUS imaging reflected the cyst wall, which was visible as the contrast agent passed through the microvessels. The hypo-enhancement within the cystic wall is due to sparse blood vessels, resulting in a low density of contrast microbubbles visualized as a low-intensity microbubble reflection. The absence of enhancement was due to the absence of perfusion of contrast material within the mucin component. These features are considerably different from the sonographic picture of splenic infarction, which is generally wedge-shaped, with the tip pointing to the splenic hilum and no obvious contrast agent entering the infarct area (24).

* In conclusion, this article summarizes the 2D and CEUS findings of LAMN, a differential diagnosis for septate mixed cystic-solid echo in the right lower abdomen of a female patient. Meanwhile, physical examination findings should always be correlated such that the mass moves in the opposite direction to the ovary. CEUS also allows analysis of blood flow within a tumor without the need for radiation exposure as in angiography. Since LAMN lesions may metastasize to abdominal organs, clinicians should look for corresponding ultrasound manifestations as well.

## Author contributions

MC and YX contributed substantially to the conception and design of the study. MC and YX contributed substantially to the acquisition of the clinical and imaging data. MC and SZ participated in literature review. MC and YX developed the first draft of the manuscript, which was reviewed and revised by YS. All authors contributed to the article and approved the submitted version.

## References

[1] Carr NJ Cecil TD Mohamed F Sobin LH Sugarbaker PH González-Moreno S. A consensus for classification and pathologic reporting of pseudomyxoma peritonei and associated appendiceal neoplasia: the results of the Peritoneal Surface Oncology Group International (PSOGI) modified Delphi process. Am J Surg Pathol (2016) 40:14–26. doi: 10.1097/PAS.0000000000000535 26492181

[2] Carr NJ McCarthy WF Sobin LH. Epithelial noncarcinoid tumors and tumor-like lesions of the appendix. A clinicopathologic study of 184 patients with a multivariate analysis of prognosticfactors. Cancer (1995) 75:757–68. doi: 10.1002/1097-0142(19950201 7828125

[3] Tirumani SH Fraser-Hill M Auer R Shabana W Walsh C Lee F. Mucinous neoplasms of the appendix: a current comprehensive clinicopathologic and imaging review. Cancer Imaging (2013) 13(1):14–25. doi: 10.1102/1470-7330.2013.0003 23439060 PMC3582328

[4] Carr N Sobin L. Tumors of the appendix. In: Bosman FT Carneiro F Hruban RH Theise ND, editors. WHO Classification of tumours of the digestive system. World Health Organization classification of tumours vol 3. 4th. Lyon, France: IARC Press (2010). p. 122–25.

[5] Borges AL Carvalho C Chorão M Pereira H Djokovic D. Low-grade mucinousappendiceal neoplasm mimicking an ovarian lesion: A case report and review of literature. World J Clin cases (2021) 9(10):2334–43. doi: 10.12998/wjcc.v9.i10.2334 PMC8026829 33869611

[6] Liu Z Yang F Zhang Y Yu H Zhu H Yang R. Conventional, doppler and contrast-enhanced ultrasonography in differential diagnosis of ovarian masses. Cell Physiol Biochem (2016) 39(6):2398–408. doi: 10.1159/000452508 27832653

[7] Yu XR Mao J Tang W Meng XY Tian Y Du ZL. Low-grade appendiceal mucinous neoplasms confined to the appendix: clinical manifestations and CT findings. J Investig Med (2020) 68:75–81. doi: 10.1136/jim-2018-000975 PMC6996116 31300469

[8] Padmanaban V Morano WF Gleeson E Aggarwal A Mapow BL Stein DE. Incidentally discovered low-grade appendiceal mucinous neoplasm: a precursor to pseudomyxoma peritonei. Clin Case Rep (2016) 4:1112–6. doi: 10.1002/ccr3.694 PMC5134204 27980743

[9] Xiao J Li P Liu W. Analysis of Clinical characteristics of low-grade appendiceal mucinous neoplasm (LAMN): A retrospective cohort study of 51 LAMN patients. J Invest Surg (2020) 34(7):1–7. doi: 10.1080/08941939.2019.1695986 31906733

[10] Cabanas J Gomes R Zappa L Esquivel J Cerruto C Goldstein PR. Splenic metastases from mucinous neoplasms of the appendix and colon. Tumori (2006) 92(2):104–12. doi: 10.1177/030089160609200204 16724688

[11] Caspi B Cassif E Auslender R Herman A Hagay Z Appelman Z. The onion skin sign: a specific sonographic marker of appendiceal mucocele. J Ultrasound Med (2004) 23:117–21. doi: 10.7863/jum.2004.23.1.117 14756359

[12] Hajiran A Baker K Jain P Hashmi M. Case of an appendiceal mucinous adenocarcinoma presenting as a left adnexal mass. Int J Surg Case Rep (2014) 5(3):172–4. doi: 10.1016/j.ijscr.2013.12.008 PMC3955234 24568943

[13] Ploenes T Börner N Jirkpatrick C Heintz. Neuroendocrine Tumour A. Mucinous dennocarcinoma and signtr-ring cell carcinoma of the appendix: three cases and review of literature. Indian J Surg (2013) 75(Suppl 1):299–302. doi: 10.1007/s12262-012-0704-4 24426597 PMC3693327

[14] Testa AC Van Holsbeke C Mascilini F Timmerman D. Dynamic and interactive gynecological ultrasound examination. Ultrasound Obstet Gynecol (2009) 34:225–9. doi: 10.1002/uog.7309 19644933

[15] Kinoshita O Murayama Y Kuriu Y Nakanishi M Sakakura C Otsuji E. Invasive mucious adenocarcinoma associated with adjacent sessile serrated lesion of the appendix vermiform: a case report. Case Rep Pathol (2014) 2014:979674. doi: 10.1155/2014/979674 25114824 PMC4119926

[16] Mitchell A Dube P Sideris L. Dysplastic intestinal-type metaplasia of appendiceal endometriosis: a mimic of low grade appendiceal mucinous neoplasm. Diagn Pathol (2014) 21(9):39. doi: 10.1186/1746-1596-9-39 PMC4001424 24559059

[17] Pickhardt PJ Levy AD Rohrmann CA Kende AI. Primary neoplasms of the appendix: radiologic spectrum of disease with pathologic correlation. Radiographics (2003) 23:645–62. doi: 10.1148/rg.233025134 12740466

[18] Je BK Kim SB Lee SH Lee KY Cha SH. Diagnostic value of maximal-outer-diameter and maximal-mural-thickness in use of ultrasound for acute appendicitis in children. World J Gastroenterol (2009) 15(23):2900–03. doi: 10.3748/wjg.15.2900 PMC2699009 19533813

[19] Koh JL Liauw W Chua T Morris DL. Carbohydrate antigen 19-9 (CA 19-9) is an independent prognostic indicator in pseudomyxoma peritonei post cytoreductive surgery and perioperative intraperitoneal chemotherapy. J Gastrointestinal Oncol (2013) 4(2):173–81. doi: 10.3978/j.issn.2078-6891.2012.062 PMC3635178 23730513

[20] Dulskas A Poskus T Poskus E Strupas K. Long-term outcomes after surgery for appendiceal mucinous tumours. Visceral Med (2018) 34(2):151–5. doi: 10.1159/000485092 PMC5981675 29888246

[21] Yoshida Y Sato K Tada T Maekawa H Sakurada M Orita H. Two cases of mucinous cystadenoma of the appendix successfully treated by laparoscopy. Case Rep Gastroenterol (2013) 7(1):44–8. doi: 10.1159/000346299 PMC3573785 23467399

[22] Dachman A Lichtenstein J Friedman A. Mucocele of the appendix and pseudomyxoma peritonei. Am J Roentgenol (1985) 144:923–9. doi: 10.1159/000346299 3885692

[23] Neesse A Huth J Kunsch S Michl P Bert T Tebbe JJ. Contrast-enhanced ultrasound pattern of splenic metastases - a retrospective study in 32 patients. Ultraschall Med (2010) 31(3):264–9. doi: 10.1055/s-0028-1109812 19899027

[24] Dormagen J Meyerdierks O Gaarder C Naess P Sandvik L Klow NE. Contrast-enhanced ultrasound of the injured spleen after embolization–comparison with computed tomography. Ultraschall Med (2011) 32(5):485–91. doi: 10.1055/s-0029-1246003 21294071

